# Is the Frequent Use of Forceps Abusive

**Published:** 1889-01

**Authors:** 


BUFFALO MEDICAL AND SURGICAL JOURNAL
A MONTHLY REVIEW OF MEDICINE AND SURGERY.

EDITORS:
THOS. LOTHROP, M. D., - - - W. W. POTTER, M. D.

All communications, whether of a literary or business character, should be addressed to the editors: 284 FRANKLIN STREET, BUFFALO, N. Y

### Editorial.

### *"IS THE FREQUENT USE OF FORCEPS ABUSIVE."*

A valuable paper with the above title was read in September last before the American Association of Obstetricians and Gynecologists by Professor Opie, of Baltimore, who, with unusual force and clearness, voices the sentiments of accoucheurs of experience and skill in regard to the too frequent use in parturition of the obstetric forceps.

The rise and decline of theories in medicine, as well as of instruments in surgery, are facts observed in the history of medical science. The extreme conservatism of the older writers, as Hunter, Denman, Osborn, etc., who trusted labor almost entirely to nature, has given place, with the lapse of time, to the opposite extreme, in which nature is supplanted by art in the completion of the parturient effort by the too general use of instruments.

Professor Opie emphasizes the principle that nature, when at her relative norm in childbirth, provides a vital power, precise in amount and aim for the varying needs of the individual, and when at times she seems greatly at fault, her deficiencies may be wisely supplemented by the accoucheur, who is presupposed to possess a thorough knowledge of the anatomy of the pelvis, of the fetal head, and of the physiological and mechanical phenomena of labor. The opportunities for the student to acquire more than a theoretical knowledge of obstetrics, he regrets, have been until recently very limited. The instruction in this important department of medical science being chiefly didactic and rarely clinical, is responsible for the vagaries prevailing in the profession, especially in the use and abuse of instruments in labor. This deficiency is now recognized, and has been corrected in many of our medical schools, notably in the establishment of the Maternity in Baltimore, which is under the professional supervision of Dr. Opie, thus affording him the opportunity to inculcate at the bedside the principles advocated in this excellent paper.

We may say with pride that the didactic lectures on obstetrics, in the Medical Department of Niagara University in this city, are now supplemented with clinical instruction to the senior students in the Maternity Hospital. This is the only method in which to correct faulty impressions given to the novitiate in lectures, and will restrict the application of the forceps within narrow limits.

The advocacy of the Tarnier traction-rod forceps in high operations, in that it exerts traction in the right direction, and requires the exercise of less force, and affords greater safety to both mother and child, we desire to warmly endorse; while the necessity of utilizing all the vis-a-tergo and only enough of the operator's own strength to bring the combined forces to the norm, and all above that should be reckoned as *abuse* are principles in clinical obstetrics, which are wise and practical, and should be adopted by every conscientious obstetrician. Charpentier's maxim is endorsed, that among the qualities of the medical man is "to know how to wait patiently," and that a watchful expectancy would give better results than "pernicious activity."

The statistical data furnished by the essayist, and the conclusion drawn from them, are pertinent to the subject under discussion. For instance, in twelve years (1863 to 1875) in Guy's Hospital, the forceps-rate was 1 in 200 deliveries; the still-births, 2.7 per cent. In St. Thomas's, in 1874, the forceps-rate was 1 in 18, and the still-births 2.8 per cent., not a single mother dying after forceps in this Charity. In the Rotunda Hospital, the forceps-rate from 1847 to 1875 was 16.5 per 1,000, and the maternal mortality was 13 per 1,000. From 1871 to 1875 the forceps-rate was 96.4 per 1,000, and the mortality 22 per 1,000, the latter high mortality being attributed to the prevalence of puerperal septicemia.

In St. Thomas's Charity, in 1874, the forceps-rate was 54.2 per 1,000, and the mortality 7.4 per 1,000. In 1875 the forceps-rate was 61.8 per 1,000, and the mortality 3.4 per 1,000, the mean mortality being 5.4 per 1,000.

These statistics, like all data without the concomitant conditions, are simply guides in forming an estimate of the influence of instrumental interference as a factor in the causation of maternal and fetal mortality.

The most valuable data is furnished from the Baltimore Maternity from 1874 to 1888 inclusive. During this period there were 1495 labors, in which the forceps-rate was 9.4 per cent., with only two deaths, one of which was from septicemia. What influence the use of the forceps exerted in the latter case, the writer does not state. In the last 100 cases of labor, the forceps were applied only twice, with no sepsis resulting, and one death. Professor Opie concludes with the statement that one in fifteen to one in twenty labors is as often as forceps need be used. We think the rate may be restricted yet more with safety to both mother and child.

This paper is the most valuable and clearest exposition of the important subject of forceps' use and abuse we have perused, and we congratulate the writer for directing attention to the subject, and for the experience and data with which he sustains his position. Such a production is opportune, and will exert a wide influence among medical men, who, like-minded, regard a wise conservatism in the use of instruments in labor to be an essential auxiliary in relieving the pain and perils of childbirth, and in lessening maternal and fetal mortality.

---

THE MEDICAL ANALECTIC has put on a new cover. We always liked this journal. We like it all the better in its more substantial dress.

We are gratified to announce that Dr. A. M. Ewing, a graduate of Trinity University, Toronto, and a member of the Royal College of Surgons, England, has consented to give a course of lectures on Pathology at the Medical Department of Niagara University in this city, the lectures extending over the last half of the present session. Dr. Ewing has enjoyed exceptional advantages in this department, and comes to our city with the highest testimonials as to his ability and professional acquirements. We bespeak for the new lecturer the success in his work which is the result of earnest labor and genuine enthusiasm in a most important branch of medical study, and we feel assured that both the college and the profession in this city will find in him an assiduous student, as well as a valuable addition to the increasing number of educated and accomplished physicians.

The Canadian Practitioner, one of the handsomest of our exchanges, announces that, with the beginning of the new year, it will be published as a semi-monthly, instead of a monthly, as heretofore. We congratulate our Toronto contemporary upon a prosperity that warrants such an important change, and we wish it the continued success it so well merits, under the new departure.

